# The Impact of Competitive and Collaborative Environments on Vocational Students’ Competitive Attitudes, Task Motivation, and Adaptability: A Multilevel Structural Equation Modeling Analysis

**DOI:** 10.3390/bs15040433

**Published:** 2025-03-28

**Authors:** Cheng Ma, Bo-Ching Chen

**Affiliations:** 1 Department of Physical Education, University of Shanghai for Science and Technology, Shanghai 200093, China; 2 Undergraduate Program of Sports Coaching, CTBC Business School, Tainan 709301, Taiwan

**Keywords:** career adaptability, organizational climate, learning climate, MSEM, PISA

## Abstract

With the rapid changes in external environments, cognitive adaptability has become crucial for vocational students’ personal growth and career development. However, previous research has predominantly focused on traditional single-level effects, overlooking the multilevel impacts of school climates. Hence, based on social cognitive theory and social–ecological systems theory, this study employs multilevel structural equation modeling (MSEM) to examine the effects of competitive and collaborative environments on vocational students’ competitive attitude, task motivation, and cognitive adaptability at both the student level (Within) and school level (Between). This study utilizes data from the Programme for International Student Assessment (PISA) 2018, analyzing a sample of 814 vocational schools and 20,978 vocational students from 18 countries and regions. Using Mplus 8.10, we applied maximum likelihood estimation with robust standard errors (MLR) to validate the multilevel structural equation model (MSEM) and examine the hierarchical effects of competitive and collaborative environments on vocational students’ competitive attitude, task motivation, and cognitive adaptability. The findings indicate that both competitive attitude and task motivation positively impact cognitive adaptability at both the student and school levels. While competitive environments enhance competitive attitudes at both levels, their effects on task motivation differ, as they are positive at the student level but negative at the school level. Conversely, collaborative environments positively influence task motivation at both levels but only affect competitive attitudes at the student level. A comparison between multilevel and single-level models suggests that multilevel modeling better captures the hierarchical effects within school environments. The results highlight that moderate competition at the student level fosters motivation and adaptability, whereas highly competitive school environments may suppress motivation. In contrast, fostering a collaborative school climate enhances task motivation and cognitive adaptability. These findings underscore the importance of balancing competition and collaboration in vocational education to support students’ holistic development.

## 1. Introduction

Vocational schools serve as a pathway to enhance professional skills, playing a significant role in increasing students’ job prospects (Horanicova et al., 2024). Compared with traditional schools, students in vocational institutions focus more on how to develop practical skills through learning, and the learning goals are generally oriented toward directly entering the labor market (He et al., 2023). However, with advancements in society, technology, and digital transformation, the complexity and uncertainty of the employment environment have intensified. The ability to adapt effectively to a constantly changing external environment has become a critical determinant of vocational students’ personal development and career growth. Consequently, cultivating vocational students’ cognitive adaptability in such a dynamic context is of paramount importance. Cognitive adaptability refers to a student’s ability to dynamically adjust their thinking and behaviors to adapt to unpredictable and changing environments (Beaudoin et al., 2024). This capability is crucial for students to effectively address challenges and achieve personal growth (Chuang et al., 2022; Zhou & Lin, 2016). Previous studies suggest that cognitive adaptability is influenced by individuals’ ability to regulate their cognition, behavior, and emotions in uncertain and ever-changing environments (Waldeck et al., 2021). However, the underlying mechanisms of cognitive adaptability remain underexplored, particularly in the context of vocational education. How school environments and personal factors can foster students’ cognitive adaptability is still unclear.

Previous studies have investigated cognitive adaptation based on the social cognitive theory and demonstrated that cognitive adaptability is closely associated with personal factors, including attitudes, self-efficacy, and motivation (de-la-Peña et al., 2021; Q. Wang et al., 2022; S. Yang & Pu, 2022). Additionally, this theory also underscores the influence of external environments on individual cognition and behavior, as well as the interaction between environments, cognition, and behavior (Yin, 2022). However, most studies have explored the impact of individual factors on cognitive adaptability within a single ecological level (X. Li et al., 2022; S. Yang & Pu, 2022). Social–ecological systems theory posits that individual behavior is shaped by the interactions of multilevel environmental systems (Bronfenbrenner, 1979). Schools, formed by creating the culture and establishing regulations, represent a mesosystem and play a crucial role in students’ development; moreover, students’ subjective perceptions of the campus atmosphere are part of the microsystem, and this atmosphere influences their attitudes, cognition, and behavior (Ehrenreich et al., 2012).

The school environment primarily consists of competition and cooperation (Ma & Chen, 2024). Few studies have systematically examined how school environments (such as competitive and collaborative atmospheres) and personal factors (such as competitive attitude and task motivation) influence vocational students’ cognitive adaptability from a multilevel ecological systems perspective.

The aims of this study are to fill the research gap and capture the nuanced impacts of personal and environmental factors at multiple levels by constructing a multilevel analytical framework integrating social cognitive theory and social–ecological systems theory. Using multilevel structural equation modeling (MSEM), this study distinguishes between “student level (Within)” and “school level (Between)” to examine the effects of competitive attitude, task motivation, and school environments (competition and collaboration) on cognitive adaptability. By systematically analyzing how competitive and collaborative environments within the microsystem and mesosystem influence students’ task motivation and competitive attitudes, this study aims to address gaps in existing literature on multilevel educational environment dynamics. Furthermore, the study provides theoretical insights for optimizing educational systems. Based on social cognitive theory, this research also integrates motivation, attitude, and behavioral regulation models with the multilevel perspective of social–ecological systems theory to explore how personal factors (motivation and attitude) influence cognitive adaptability. It examines potential differences across school-level systems. Through this systematic analysis, the study seeks to reveal the roles of task motivation and competitive attitude in enhancing vocational students’ cognitive adaptability, offering theoretical and practical implications for fostering adaptability in vocational education.

Based on the above, the research questions are as follows:

1. How do competitive attitude and task motivation, as individual cognitive factors, influence vocational students’ cognitive adaptability?
2. Do competitive and collaborative school environments exert differentiated effects on the development of cognitive adaptability?
3. How do traditional single-level models compare with multilevel models in explaining the aforementioned relationships?

Based on the research background and questions outlined above, this study requires a large sample of vocational school students at both the within-group and between-group levels to ensure the representativeness and generalizability of the findings. The PISA 2018 student dataset aligns well with the research scope and objectives, making it an appropriate data source for this study. The PISA (Programme for International Student Assessment) is a large-scale international assessment developed by the Organisation for Economic Co-operation and Development (OECD). Since 2000, it has been conducted every three years to evaluate 15-year-old students’ competencies in reading, mathematics, and science. The assessment follows a standardized sampling procedure, selecting students randomly within participating countries to ensure representativeness and cross-national comparability. Therefore, this research adopts a secondary data analysis approach and applies multilevel structural equation modeling (MSEM) to account for the hierarchical nature of the data. Due to space limitations, detailed characteristics of vocational school students in different countries can be found in the PISA 2018 Technical Report (OECD, 2019a).

## 2. Theoretical Foundation and Research Hypotheses

### 2.1. Social Cognitive Theory: Relationships Between Motivate, Attitude, and Adapt

#### 2.1.1. Cognitive Adaptability in Educational Context

Cognitive adaptability (Adapt) originates from psychology but has been widely applied in fields such as education and management. It refers to a student’s ability to dynamically and flexibly adjust their thinking and behavior to adapt to new and unpredictable circumstances (Moore & Wang, 2017). In the context of education, students with higher cognitive adaptability show a higher ability to adapt to the changing environment stimuli with divergent thinking and creativity (Begum et al., 2023; Xia et al., 2022). This concept emphasizes the proactive capacity of students to navigate environmental changes and achieve their goals (Xing et al., 2022). In the context of the 2018 Programme for International Student Assessment (PISA), cognitive adaptability is defined in the field of education as vocational students’ ability to adjust their thinking and behavior to adapt to mainstream cultural environments or challenging new situations. Students with high cognitive adaptability are better equipped to manage stress and negative emotions, such as frustration, in demanding contexts. They also demonstrate resilience and possess broader perspectives for problem solving (OECD, 2020). Research indicates that cognitive adaptability is closely linked to both academic and non-academic outcomes, playing a pivotal role in students’ academic development and personal achievements (Levin, 2015; Martin et al., 2013). Thus, cognitive adaptability underscores the ability to respond effectively to environmental changes and is closely associated with vocational students’ psychological resilience and cognitive flexibility. Psychological resilience is strongly correlated with well-being, as shown in previous research; it involves the ability of students to positively cope with difficulty and promotes adaptation and development in demanding conditions (Bajaj & Pande, 2016; Tan et al., 2021). These traits are critical factors in enhancing both their academic performance and personal accomplishments.

#### 2.1.2. The Relationship Between Adapt, Motivate, and Attitude Based on Social Cognitive Theory

Social cognitive theory posits that learning and behavioral changes are the result of interactions between cognitive factors, behavioral patterns, and environmental influences (Bandura, 1989). According to this framework, cognitive adaptability is shaped not only by external environmental and behavioral factors but also by individual factors (Schunk & DiBenedetto, 2020). This study conceptualizes competitive attitude and task motivation as personal factors. Specifically, students who exhibit higher levels of competitiveness and motivation are expected to demonstrate greater cognitive adaptability. It is therefore important to examine whether a significant relationship exists among competitive attitude, task motivation, and cognitive adaptability in vocational school students. Grounded in Social Cognitive Theory, this study investigates how these personal factors contribute to the development of cognitive adaptability.

Competitive Attitude (Attitude): Competitive attitude reflects a student’s perceptions and tendencies regarding competition (H. Wang et al., 2018). As one dimension of adolescents’ social interdependence attitudes, it plays a vital role in vocational students’ motivation and academic performance within learning environments (Lee & Seo, 2022). Several studies have shown that competitive attitudes have an impact on psychological well-being, self-esteem, and task motivation, and one of them has found that the competitive attitude can increase task motivation among vocational students (Ding et al., 2023; Ma & Chen, 2024). The underlying mechanism can be inferred from previous research, which demonstrated that students with a highly competitive attitude often exhibit a performance-approach orientation. These students tend to engage actively in competitive scenarios and strive to outperform their peers. This achievement orientation has been shown to enhance self-efficacy (Kool et al., 2016). According to Social Cognitive Theory, self-efficacy is the strongest predictor of motivation (Conradty & Bogner, 2022).

Moreover, the competitive attitude may be correlated with exploring the environment and seeking a way to succeed (Nikolašević et al., 2025). One study has implied that individuals with a highly competitive attitude would show better performance in the workplace and secure better career development; that is, they will adopt proactive cognitive strategies and seek optimal solutions even when faced with challenges or complex situations so that they adapt to the external environment and achieve better job performance (H. Wang et al., 2018). In the education field, teenagers’ competitive attitude is a very important factor for social adaptability, which means it is a force to push students toward the process of self-exploring and growth (Ding et al., 2023). Since cognitive adaptability entails the ability to adjust thinking and behaviors flexibly to navigate novel circumstances, it is closely related to this positive psychological process. Hence, this study proposes the following hypotheses:

**H_1_:**  *Competitive attitude positively influences cognitive adaptability.*

**H_2_:**  *Competitive attitude positively influences task motivation.*

Task Motivation (Motivate): Motivation, a core concept in social cognitive theory, is an intrinsic driving force that explains individual behavioral patterns (Schunk & DiBenedetto, 2020; Chuang et al., 2022). In line with the motivational emphasis in social cognitive theory, the PISA 2018 highlights task motivation as a cognitive process that initiates and sustains goal-directed activities (Schunk & DiBenedetto, 2020). Task motivation is essential for fostering cognitive adaptability, as it encourages students to employ self-regulatory strategies to adjust their behaviors and cognitive approaches, enabling them to tackle novel and complex challenges effectively (Abdelrahman, 2020; Ito & Umemoto, 2022; Naamati-Schneider & Alt, 2023). Hence, this study proposes the following hypothesis:

**H_3_:**  *Task motivation positively influences cognitive adaptability.*

Through the lens of social cognitive theory, this study examines how individual factors such as competitive attitude and task motivation interact with cognitive adaptability, thereby contributing to a deeper understanding of the mechanisms that promote flexibility and resilience in vocational students.

### 2.2. Social–Ecological Systems Theory: Exploring the School Environment’s Role

The social–ecological systems theory, introduced by Bronfenbrenner, posits that individual behavior and development are influenced by the interactions between the individual and their surrounding environment across multiple levels (Bandura, 1989; Bronfenbrenner, 1979). This study adopts the social–ecological systems framework, embedding individual factors of student development within interconnected environmental systems. It investigates how the school environment influences vocational students’ task motivation and competitive attitude, using multilevel analysis to identify the differential impacts of competitive and collaborative school environments across various levels.

According to social–ecological systems theory, studying individual development requires an ecological framework, as personal growth is shaped not only by individual factors but also by environmental influences, representing a dynamic response to the surrounding context (Bronfenbrenner, 1977). In the context of vocational education, task motivation and competitive attitude are influenced not only by students’ personal cognition and their subjective perceptions of the environment (student level) but also by broader organizational factors at the school level.

The school environment, including its competitive or collaborative atmosphere, functions as a mesosystem within the educational structure and forms an essential component of the social–ecological system. This study applies this theoretical framework to capture the dynamic interactions between the microsystem (students’ subjective perceptions of their environment) and the mesosystem (the organizational climate of schools). By doing so, it aims to provide a more comprehensive understanding of how competitive and collaborative school environments influence vocational students’ task motivation and competitive attitudes.

### 2.3. Influence of the Competitive Environment (CompEnv)

#### 2.3.1. The Influence of Competitive Environment on Competitive Attitude

Competitive attitude, which reflects a student’s subjective preference for competition, is shaped by external environmental factors such as the competitive environment. As an abstract organizational variable, competitive environment represents the intensity and characteristics of competition within the school culture, including aspects like resource scarcity, incentive mechanisms, and social norms (Delay & Piou, 2019; Ma & Chen, 2024; Sun et al., 2020). A recent study has shown that the competitive environment may positively influence students’ attitudes toward competition in the vocational education setting (Ma & Chen, 2024). The influence of competitive environment on competitive attitude operates through two primary mechanisms. First, the social comparison mechanism fosters an atmosphere where individuals compare their performance with others, stimulating a sense of competition and pressure (H. Wang et al., 2018). For instance, when individuals perceive that their abilities provide a competitive advantage, they are more likely to engage in competition. Second, the goal orientation enhancement mechanism highlights how intense competitive scenarios introduce challenges and opportunities, reinforcing competitive attitudes through external rewards and punishments, thereby fostering clearer performance-oriented goals (Mayor et al., 2020). Together, these mechanisms strengthen vocational students’ competitive attitudes in competitive environments. Based on these findings, the study proposes the following hypothesis:

**H_4_:**  *The competitive environment positively influences competitive attitude.*

#### 2.3.2. The Influence of Competitive Environment on Task Motivation 

Social cognitive theory emphasizes that human behavior results from the interaction between cognition and the external environment (Bandura, 1989). As a key environmental variable within school culture, the competitive atmosphere affects students’ task motivation and academic performance (Ma & Chen, 2024). However, in recent decades, teaching practices that encourage a competitive learning atmosphere have been a contentious topic in the field of education (Ooi & Cortina, 2023).

On one hand, some studies argue that competitive learning environments enhance students’ task motivation and academic performance (Lamb et al., 2019). On the other hand, other research finds that highly competitive school cultures and strict rules can reduce students’ independence and autonomy, thereby decreasing their willingness to take on challenging tasks (Ooi & Cortina, 2023; Tan et al., 2023). Furthermore, while competitive school environments may help students with high self-efficacy improve their academic performance (Tan et al., 2023), excessive emphasis on competition can hinder academic development and mental health for others (Ooi & Cortina, 2023).

These findings suggest that the competitive atmosphere within school culture influences students’ task motivation. However, the direction and intensity of this influence remain controversial. This study seeks to address this gap by applying a multilevel perspective based on social–ecological systems theory to analyze the effects of competitive environments on vocational students’ task motivation. Accordingly, the following hypothesis is proposed:

**H_5_:**  *The competitive environment positively influences task motivation.*

### 2.4. Influence of the Collaborative Environment (CollEnv)

#### 2.4.1. The Influence of Collaborative Environment on Competitive Attitude

The collaborative environment is a crucial component of school culture that complements the competitive environment. It emphasizes teamwork, resource sharing, and positive interactions (Huang et al., 2024; F. Wang et al., 2024). Within the field of education, the collaborative environment is widely regarded as a positive learning atmosphere that enhances academic performance, self-efficacy, and student engagement (F. Wang et al., 2024). Through mutual support and teamwork, collaborative environments can influence the development of students’ competitive attitudes and task motivation (Ooi & Cortina, 2023; Qian et al., 2021).

Despite numerous research studies on collaborative environments, the impact of vocational schools’ collaborative climate on competitive attitude is still unknown. However, some clues can be inferred from previous research, and it is worth emphasizing that the influence of a collaborative environment on students’ competitive attitudes might exhibit variability across different levels. At the microsystem level, social cognitive theory suggests that individuals’ cognition and attitudes are driven by subjective perceptions of their external environment (Bandura, 1986). When students perceive a cooperative atmosphere within the campus, positive interpersonal interactions can alleviate the tension and threat often associated with competitive situations. This redefines competition as a constructive and engaging interpersonal experience, potentially fostering a more positive competitive attitude among students (Ooi & Cortina, 2023).

Additionally, from a mesosystem perspective, social–ecological systems theory (Bronfenbrenner, 1979) posits that behavior results from interactions between microsystems (individual perceptions), mesosystems (school and classroom environments), and macrosystems (social norms and educational systems). The school’s collaborative environment, as a key mesosystem element within the education system, provides a structured setting for fostering teamwork and building positive interpersonal relationships. Such an environment plays a pivotal role in individual development (Chen et al., 2024). Although previous research has highlighted the importance of collaboration for personal growth, academic achievement, and mental health, few studies have explored the mechanisms through which a mesosystem-level collaborative atmosphere influences students’ competitive attitudes, particularly its multilevel effects across educational systems.

This study, grounded in social cognitive theory and incorporating a multilevel perspective from social–ecological systems theory, investigates the differential impacts of collaborative environments on vocational students’ competitive attitudes. To address the existing research gaps, the following hypothesis is proposed:

**H_6_:**  *The collaborative environment positively influences competitive attitude.*

#### 2.4.2. The Influence of Collaborative Environment on Task Motivation

Motivation is the foundation of willpower and capability and a critical determinant of academic performance (Chuang et al., 2022). In educational contexts, the role of the environment in shaping and fostering student motivation is vital. As Kaplan and Patrick (2016) noted, education essentially involves designing learning environments to stimulate students’ motivation and engagement. Collaborative learning environments encourage students to cooperate, share resources, and work toward common learning goals. This process not only improves academic outcomes but also significantly enhances students’ motivational levels (C. Yang et al., 2021).

Goal orientation theory distinguishes between learning goals (also referred to as mastery goals) and performance goals (Stasielowicz, 2019). Mastery goals focus on the intrinsic value of the learning process and are closely tied to students’ internal interest and learning strategies (Jang & Lee, 2021; Telyani et al., 2021). Research has demonstrated that collaborative learning can foster students’ mastery goal orientation, which is strongly linked to intrinsic motivation. This motivation helps address students’ lack of drive in learning and positions collaborative environments as effective contexts for enhancing task motivation (Liu & Lipowski, 2021; Mendo-Lázaro et al., 2021).

Based on this, the following hypothesis is proposed:

**H_7_:**  *The collaborative environment positively influences task motivation.*

### 2.5. The Conceptual Model

**Figure 1:**
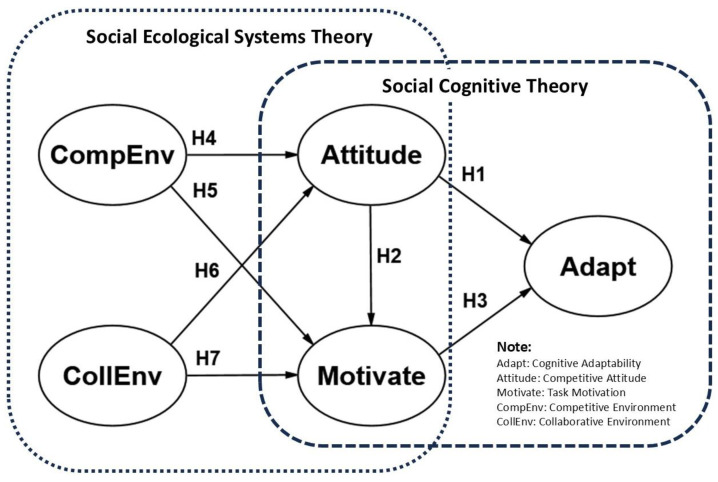
Research model.

Based on the literature review and research hypotheses, this study develops a conceptual model (as shown in Figure 1 and Table 1) integrating social cognitive theory and social–ecological systems theory. The model examines the multilevel effects of competitive and collaborative school environments on vocational students’ cognitive adaptability, task motivation, and competitive attitude, addressing both student-level (Within) and school-level (Between) influences. This framework provides a basis for testing the proposed relationships through multilevel structural equation modeling (MSEM).

## 3. Methodology

This study employs a secondary data analysis approach, utilizing the PISA 2018 (Programme for International Student Assessment) dataset (available at: https://www.oecd.org/en/data/datasets/pisa-2018-database.html (accessed on 7 February 2023).). The PISA adopts a two-stage multilevel sampling strategy: first, schools are randomly selected, and then students are randomly sampled within the selected schools (Molina-Muñoz et al., 2023; Y.-J. Wang & Chen, 2023). While this sampling method ensures the representativeness of the dataset, it also introduces a nested structure, where students are nested within schools. This violates the assumption of sample independence, thereby justifying the use of multilevel structural equation modeling (MSEM) in this study.

From this multilevel perspective, the study incorporates both student-level and school-level variables to explore the pathways through which competitive and collaborative environments influence vocational students’ attitudes, motivation, and adaptability. The following sections detail the specific steps and procedures of the research methodology.

### 3.1. Participants

**Figure 2:**
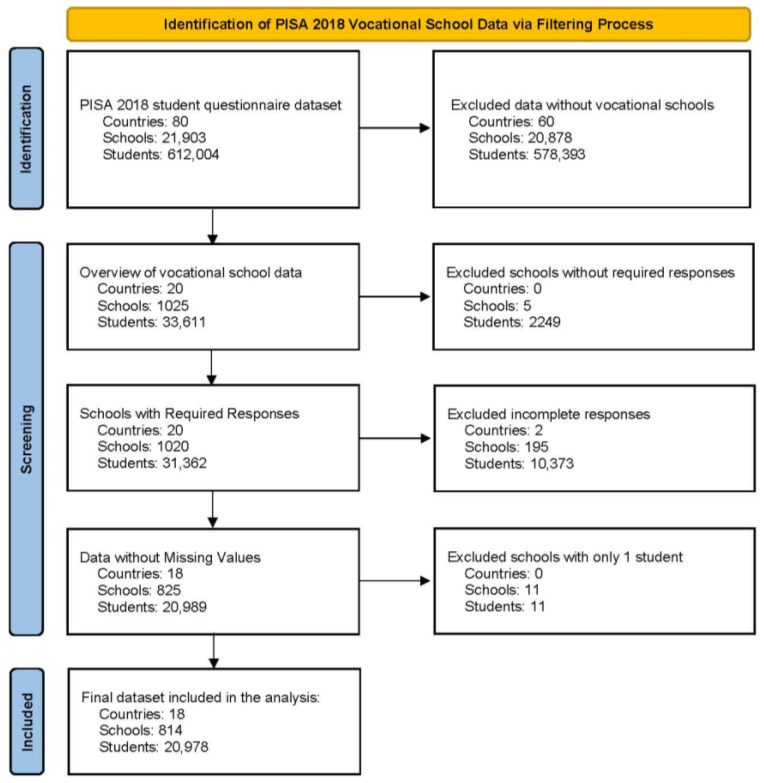
Data selection and filtering process for PISA 2018. Note: The design of this figure was inspired by the structured approach to data filtering visualization, as commonly used in systematic review studies (e.g., Page et al., 2021). However, the dataset selection criteria and implementation were independently developed based on the characteristics of PISA 2018.

This study analyzed data from the PISA 2018 student questionnaire database, which originally included 612,004 students from 21,903 schools across 80 countries. To clearly present the data selection process, a PRISMA-style flow diagram, commonly used in systematic reviews (Page et al., 2021), was employed to outline the detailed data filtering steps (as shown in Figure 2). Through a systematic selection process, the dataset was initially narrowed down to countries with vocational education schools, reducing the sample to 33,611 students from 1025 schools across 20 countries. Subsequently, schools and data with incomplete responses to the study-relevant items were excluded, along with schools that had responses from only one student, to avoid the absence of variability at the organizational level. The final sample included 20,978 vocational students from 814 schools across 18 countries. This rigorous and systematic selection process ensured the completeness and representativeness of the data for vocational education institutions, making it suitable for multilevel analysis. For further details on the characteristics of vocational school students across different countries, readers may refer to the PISA 2018 Technical Report (OECD, 2019a, 2019b, 2020).

### 3.2. Measures

The study utilized items from the PISA 2018 student questionnaire to assess key variables of interest, including cognitive adaptability (6 items), competitive attitude (3 items), task motivation (4 items), perceived competitive environment (4 items), and perceived collaborative environment (4 items). Notably, cognitive adaptability was measured on a 5-point Likert scale, while all the other variables used a 4-point scale. To account for this measurement variation, standardized coefficients were applied in the MSEM analysis, effectively mitigating potential scale-related bias (Schweizer, 2011). This approach aligns with the concept of quasi-scale invariance (Wen et al., 2010), ensuring that parameter estimates remain comparable across different measurement scales while maintaining the interpretability and robustness of the results. Details of these measures are provided below.

#### 3.2.1. Cognitive Adaptability (Adapt)

This construct measures vocational students’ ability to adapt to new situations and solve challenges, reflecting their flexibility in adjusting thoughts and behaviors in diverse environments. It includes skills such as managing adverse situations and adapting to cross-cultural contexts (Chuang et al., 2022; OECD, 2020; Tomul et al., 2024). Example items include: “*I can adjust my behavior to meet the demands of new situations*” and “*I can easily adapt to new cultures*”. Responses are rated on a 5-point Likert scale (1 = Not at all like me, 5 = Very much like me). After reverse scoring, higher scores indicate stronger cognitive adaptability among vocational students.

#### 3.2.2. Competitive Attitude (Attitude)

This scale captures students’ behavioral orientation and psychological state in competitive environments, reflecting their tendency to seek self-improvement through comparison and interaction with others (Eriksson & Strimling, 2023; Ma & Chen, 2024; Menesini et al., 2018). Example items include: “I enjoy working in situations involving competition with others” and “I try harder when I am competing with others”. Responses are measured on a 4-point Likert scale (1 = Strongly disagree, 4 = Strongly agree), with higher scores indicating a more positive competitive attitude.

#### 3.2.3. Task Motivation (Motivate)

Task motivation assesses students’ engagement and persistence in the learning process, including their intrinsic drive and problem-solving abilities (Chuang et al., 2022; Ma & Chen, 2024; OECD, 2019b). Example items include: “*When I start a task, I persevere until I finish*” and “*I feel satisfied when I perform better than before*”. Responses are rated on a 4-point Likert scale (1 = Strongly disagree, 4 = Strongly agree), with higher scores indicating greater motivation to accomplish academic tasks.

#### 3.2.4. Perceived Competitive Environment (CompEnv)

This measure captures students’ perceptions of the competitive atmosphere within their schools, including the importance and prevalence of competitive behaviors among peers (Ma & Chen, 2024; OECD, 2019b; Ooi & Cortina, 2023; Y.-J. Wang & Chen, 2023). Example items include: “*Students seem to value competition*” and “*It feels important to compete with peers*”. Responses are collected on a 4-point Likert scale (1 = Strongly disagree, 4 = Strongly agree), with higher scores indicating stronger perceptions of a competitive school environment.

#### 3.2.5. Perceived Collaborative Environment (CollEnv)

This scale evaluates students’ perceptions of the collaborative atmosphere within their schools (OECD, 2019a; Ooi & Cortina, 2023; Y.-J. Wang & Chen, 2023). Example items include: “*Students seem to collaborate with each other*” and “*Students feel that cooperation is very important*”. Responses are rated on a 4-point Likert scale, with higher scores reflecting stronger perceptions of a collaborative school environment.

Details of all the items and their corresponding variables are presented in Appendix A.

### 3.3. Data Analysis

This study employed multilevel structural equation modeling (MSEM) for data analysis, using the Mplus 8.10 statistical software and the maximum likelihood estimation with robust standard errors (MLR) method for parameter estimation. To account for the PISA’s complex sampling design, we incorporated appropriate sampling weights (WEIGHT = W_FSTUWT), clustering (CLUSTER = SCID), and stratification (STRATIFICATION = STRATUM) in our analyses, ensuring unbiased parameter estimates and correct standard errors that properly reflect the multilevel structure of the data. Further details regarding the Mplus syntax can be found in Appendix D.

As an applied empirical study, the analysis procedure was guided by two key methodological frameworks: the five-step MSEM process proposed by Muthén (1989, 1994) and the traditional two-step SEM approach by Anderson and Gerbing (1988). To enhance efficiency and practicality, the study simplified the process into two main stages: measurement model validation and structural model testing. However, if issues such as model non-convergence occurred during the MSEM analysis, the research team systematically revisited the original five-step MSEM process to diagnose and address the issues. This iterative strategy ensured both the methodological rigor and practical relevance of the statistical analyses. The details of the statistical validation process are outlined below.

#### 3.3.1. Single-Level and Multilevel Confirmatory Factor Analysis (MCFA)

Following the two-step approach by Anderson and Gerbing (1988), the study conducted single-level confirmatory factor analysis (CFA) and multilevel confirmatory factor analysis (MCFA) to validate the measurement models. This included calculating the composite reliability of latent variables within each model and examining their convergent validity and discriminant validity (Fornell & Larcker, 1981).

Additionally, intraclass correlation coefficients (ICC) were computed to evaluate the degree of between-group variance, which served as a critical reference for determining whether insufficient between-group variance affected the multilevel CFA results (Cohen, 1988). If the MCFA model showed a poor fit, the ICC values provided immediate insights into whether the lack of variance was a contributing factor.

In this study, we followed the recommendations of Muthén (1989, 1994) for multilevel structural equation modeling (MSEM). Each measurement item was assigned to its respective theoretical construct, and factor loadings were freely estimated across levels. No constraints were imposed on the residual variances, allowing an unconstrained estimation of measurement error at different levels. This approach was chosen to ensure flexibility in capturing potential variations in measurement structures between the student level (Within) and the school level (Between).

#### 3.3.2. Single-Level and Multilevel Structural Model Testing (MSEM)

Following confirmation of the measurement model’s good fit (Anderson & Gerbing, 1988; Hair et al., 2009), structural model testing was conducted using both single-level SEM and multilevel SEM approaches. The single-level SEM provided a preliminary assessment of the overall fit of the variable matrix under traditional SEM, offering insights into the relationships among latent variables within the full sample. Building on these results, the multilevel SEM simultaneously analyzed the structural relationships at the student level (Within) and the school level (Between), allowing a comprehensive evaluation of the research hypotheses (Muthén, 1989, 1994) and an investigation of whether the structural relationships varied across levels. This integrated approach ensured a robust examination of the research model, capturing the nuances of its behavior across different analytical levels.

## 4. Results

This study analyzed data from the PISA 2018 database, focusing on vocational schools. The final dataset included 814 vocational schools and 20,978 vocational students from 18 countries/regions, and the analysis was conducted using multilevel structural equation modeling (MSEM). The results are presented as follows.

### 4.1. Descriptive Analysis

Among the selected vocational schools, Slovenia had the highest number of schools, with 177 schools (21.7% of the total), followed by Croatia with 104 schools (12.8%), and Hungary with 87 schools (10.7%). The vocational student sample included 20,978 students distributed across 18 countries. Slovenia had the largest number of students, totaling 2815 (13.4% of the total), followed by Croatia with 2557 students (12.2%), and Montenegro with 2414 students (11.5%). In terms of gender distribution, male students accounted for 11,478 (54.7%) of the sample, while female students accounted for 9500 (45.3%). Regarding grade levels, the largest group was 10th-grade students, totaling 12,459 (59.4%), followed by 9th-grade students with 7887 (37.6%). Smaller groups included the 8th grade with 238 students (1.1%), 11th grade with 320 students (1.5%), and 7th grade with 73 students (0.3%). Further details are provided in Appendix B.

### 4.2. Measurement Model

Following the methodology outlined earlier, single-level and multilevel confirmatory factor analyses (CFA) were conducted, along with structural model testing. The results of these analyses are presented below.

#### 4.2.1. Convergent Validity

This study assessed convergent validity based on established criteria from previous CFA research, including standardized factor loadings greater than 0.6, composite reliability (CR) above 0.7, and average variance extracted (AVE) exceeding 0.5 (Fornell & Larcker, 1981; Hair et al., 2009). As shown in Table 2, the single-level CFA analysis demonstrated that all the standardized factor loadings were above 0.60, the CR values ranged from 0.808 to 0.933, and the AVE values ranged from 0.518 to 0.777, meeting the recommended thresholds (CR > 0.70, AVE > 0.50). The model fit indices also indicated a good fit for the single-level CFA model: χ^2^(179) =1260.681, *p* < 0.001, CFI = 0.972, TLI = 0.967, RMSEA = 0.017, and SRMR = 0.021. For the multilevel CFA, the models were analyzed separately at the student level (Within) and school level (Between). At the within level, CR ranged from 0.785 to 0.923, and AVE ranged from 0.514 to 0.750. At the school level, CR ranged from 0.821 to 0.993, and AVE ranged from 0.598 to 0.973, all exceeding the recommended thresholds. The fit indices for the multilevel CFA also demonstrated an acceptable model fit: χ^2^(358) = 5938.011, *p* < 0.001, CFI = 0.968, TLI = 0.963, RMSEA = 0.027, SRMR (Within) = 0.022, and SRMR (Between) = 0.168. Furthermore, intraclass correlation coefficients (ICC) were calculated to evaluate between-group variance, with values ranging from 0.024 to 0.087. These findings confirm meaningful between-group variance, validating the appropriateness of multilevel analysis (Cohen, 1988). Overall, the results indicate good reliability and convergent validity across both the single-level and multilevel CFA models.

#### 4.2.2. Discriminant Validity

Discriminant validity was assessed using the method proposed by Fornell and Larcker (1981), which compares the square root of the AVE for each construct with the correlations between that construct and others. Discriminant validity is confirmed if the square root of the AVE exceeds the inter-construct correlations. As shown in Table 3, the single-level model results indicated that the square root of the AVE for each construct ranged from 0.720 to 0.881, all exceeding the inter-construct correlations, with the highest correlation being 0.550 (between task motivation and competitive attitude). In the multilevel model, the student-level (Within) results showed that the square root of the AVE ranged from 0.716 to 0.866 and similarly surpassed the inter-construct correlations, with the highest correlation being 0.494 (also between task motivation and competitive attitude). At the school level (Between), the square root of the AVE ranged from 0.773 to 0.986, again exceeding the inter-construct correlations, with the highest correlation being 0.683 (between competitive environment and competitive attitude). These findings demonstrate strong discriminant validity across both single-level and multilevel models, confirming the adequacy of the measurement models at both levels.

### 4.3. Structural Model Analysis

After confirming the reliability and validity of all the measurement items and constructs in both the single-level and multilevel models, the study proceeded with structural model analysis. The results are presented below.

#### 4.3.1. Model Fit

Model fit was evaluated to assess the alignment between the research model and the observed sample data (Chin, 1998). While traditional structural equation modeling (SEM) may overlook data hierarchy and risk parameter bias, it remains useful for preliminary model fit assessments (Muthén, 1994). Thus, this study reserved the presentation of traditional SEM.

As shown in Appendix C, the single-level SEM results showed a significant chi-square value (χ^2^ = 1339.685, *df* = 181, *p* < 0.001), with the fit indices demonstrating excellent fit: CFI = 0.970 and TLI = 0.965 exceeded the ideal threshold of 0.95, RMSEA = 0.017 was below the recommended 0.06, and SRMR = 0.037 was below 0.08 (Hu & Bentler, 1999; McDonald & Ho, 2002). For the multilevel SEM, the chi-square value was also significant (χ^2^ = 6216.432, *df* = 362, *p* < 0.001), and the fit indices remained strong: CFI = 0.967, TLI = 0.962, and RMSEA = 0.028, all within acceptable ranges. The SRMR for the student level (Within) was 0.033, indicating good fit, while the school level (Between) showed a less optimal SRMR of 0.159 (Hu & Bentler, 1999; McDonald & Ho, 2002). Overall, the structural model demonstrated acceptable fit for both single-level and multilevel analyses, supporting its suitability for further hypothesis testing.

#### 4.3.2. Path Analysis

**Figure 3:**
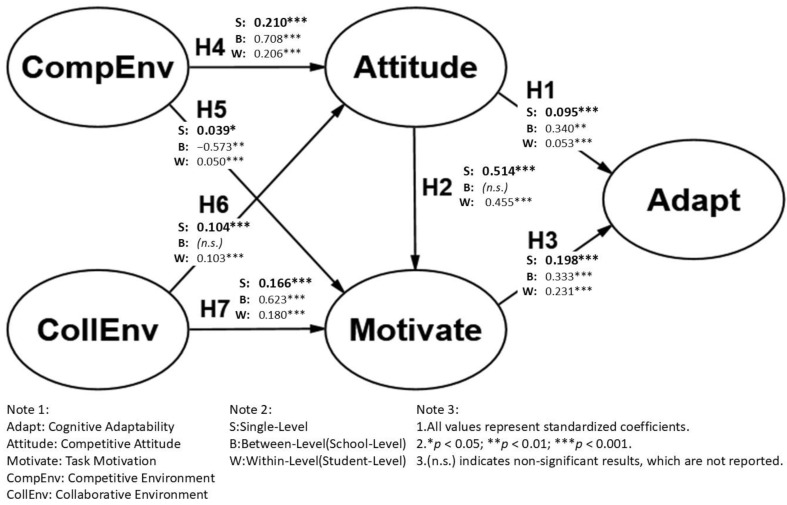
Validation of the research model.

Using multilevel structural equation modeling (MSEM), the study examined the relationships at both the student level (Within) and the school level (Between), as shown in Figure 3 and Table 4. For comparison, a traditional single-level structural equation model (SEM) was also employed to assess the differences in applicability and the results between the single-level and multilevel models. The findings for each research question are summarized below.

##### The Influence of Competitive Attitude and Task Motivation on Cognitive Adaptability

Both competitive attitude (H_1w_: *β* = 0.053, *p* < 0.001; H_1b_: *β* = 0.340, *p* < 0.05) and task motivation (H_3w_: *β* = 0.231, *p* < 0.001; H_3b_: *β* = 0.333, *p* < 0.001) had significant positive effects on cognitive adaptability at both the student and school levels, supporting H_1_ and H_3_. However, for H_2_, the positive effect of competitive attitude on task motivation was significant only at the student level (H_2w_: *β* = 0.455, *p* < 0.001) and not at the school level (H_2b_: *β* = 0.177, *p* = 0.386), indicating that H_2_ was partially supported at the student level.

##### Differential Effects of Competitive and Collaborative Environments

Competitive Environment:

The competitive environment positively influenced competitive attitude at both levels (H_4w_: *β* = 0.206, *p* < 0.001; H_4b_: *β* = 0.708, *p* < 0.001), supporting H_4_. However, the effect of the competitive environment on task motivation differed significantly between levels: it was positive at the student level (H_5w_: *β* = 0.050, *p* < 0.001) but negative at the school level (H_5b_: *β* = −0.573, *p* < 0.01).

Collaborative Environment:

The collaborative environment’s influence on competitive attitude was significant only at the student level (H_6w_: *β* = 0.103, *p* < 0.001; H_6b_: *β* = 0.146, *p* = 0.092), partially supporting H_6_. On the other hand, the collaborative environment positively affected task motivation at both levels (H_7w_: *β* = 0.180, *p* < 0.001; H_7b_: *β* = 0.623, *p* < 0.001), supporting H_7_. This suggests that a strong collaborative atmosphere can effectively stimulate students’ task motivation, particularly when supported by school-level organizational culture and institutional frameworks.

##### Comparison of Single-Level and Multilevel Model Results

The comparison between traditional single-level SEM and multilevel SEM revealed key differences across levels. At the student level, the multilevel SEM results were largely consistent with those from traditional SEM. The effects of competitive attitude and task motivation on cognitive adaptability remained stable (e.g., H_1_: *β* = 0.095, *p* < 0.001 vs. H_1w_: *β* = 0.053, *p* < 0.01; H_3_: *β* = 0.198, *p* < 0.001 vs. H_3w_: *β* = 0.231, *p* < 0.001). This suggests that single-level SEM effectively captures individual-level relationships.

At the school level, notable differences emerged. Competitive attitude and task motivation had stronger effects on cognitive adaptability (H_1b_: *β* = 0.340, *p* < 0.01; H_3b_: *β* = 0.333, *p* < 0.001). However, the relationship between competitive attitude and task motivation was no longer significant (H_2b_: *β* = 0.177, *p* = 0.386), unlike at the student level. These findings indicate that task motivation and adaptability are more strongly shaped by school-wide influences, rather than just individual traits.

When examining environmental effects, competitive environment positively influenced competitive attitude at both levels (H_4w_: *β* = 0.206, *p* < 0.001; H_4b_: *β* = 0.708, *p* < 0.001), but had opposite effects on task motivation (H_5w_: *β* = 0.050, *p* < 0.001; H_5b_: *β* = −0.573, *p* < 0.01). Collaborative environment significantly boosted task motivation at both levels (H_7w_: *β* = 0.180, *p* < 0.001; H_7b_: *β* = 0.623, *p* < 0.001), but its impact on competitive attitude was only present at the student level (H_6w_: *β* = 0.103, *p* < 0.001; H_6b_: *β* = 0.146, *p* = 0.092). These results highlight that competition at the school level may demotivate students, while collaboration fosters motivation across both levels.

Regarding the implications for model selection, while single-level SEM captures individual variations, it fails to account for school-level organizational influences. The multilevel SEM results demonstrate that the competitive environment affects task motivation differently across levels (positive at the student level, negative at the school level). Additionally, the role of competitive attitude in shaping task motivation is no longer present at the school level. Collaborative environments consistently enhance task motivation at both levels, reinforcing the role of institutional support. These differences highlight the importance of using multilevel SEM to fully understand hierarchical effects in educational settings. A detailed discussion of these findings is presented in the Discussion Section.

## 5. Discussion

Based on the findings of this study, the discussion is as follows.

### 5.1. Theoretical Considerations

This study successfully integrates social cognitive theory (Bandura, 1989) and social–ecological systems theory (Bronfenbrenner, 1979) to construct a multilevel analytical framework for understanding vocational students’ cognitive adaptability. The findings confirm the proposition that individual behavior results from an interaction between cognitive, motivational, and environmental influences, as evidenced by the significant relationships found between competitive attitudes, task motivation, and cognitive adaptability at both the student and school levels. Specifically, multilevel modeling provides empirical support for the microsystem–mesosystem interaction outlined in Bronfenbrenner’s (1979) framework, revealing how both student-level perceptions and school-level organizational climates shape cognitive adaptation. The results also reinforce social cognitive theory’s assertion that task motivation and competitive attitudes are key personal factors influencing adaptability (Schunk & DiBenedetto, 2020). This aligns with findings from Ding et al. (2023) and Ma and Chen (2024), which demonstrate the role of competition-driven motivation in shaping students’ psychological engagement. Furthermore, social–ecological systems theory explains the differential effects of competitive and collaborative environments across school levels. By extending traditional single-level SEM analyses, this study highlights the necessity of multilevel SEM in educational research, particularly in examining school-wide factors that influence student motivation and adaptation (Turner et al., 2015; Moeyaert et al., 2014).

### 5.2. Hypothesis Examination

#### 5.2.1. Cognitive Adaptation Model

The findings support H_1_ and H_3_, confirming that both competitive attitude (H_1w_: β = 0.053, *p* < 0.001; H_1b_: *β* = 0.340, *p* < 0.01) and task motivation (H_3w_: *β* = 0.231, *p* < 0.001; H_3b_: *β* = 0.333, *p* < 0.001) positively influence cognitive adaptability. This aligns with research indicating that students who embrace competition demonstrate higher adaptability (Magni et al., 2021; Menesini et al., 2018) and that task motivation enhances self-regulation and flexible learning strategies (Schunk & DiBenedetto, 2020; Abdelrahman, 2020). However, the relationship between competitive attitude and task motivation (H_2_) was significant only at the student level (H_2w_: *β* = 0.455, *p* < 0.001), but not at the school level (H_2b_: *β* = 0.177, *p* = 0.386). This suggests that students’ personal competitive attitudes drive individual motivation, but school-wide competition does not necessarily reinforce collective motivation. Prior research has noted that highly competitive school climates can create stress and reduce intrinsic motivation (Ma & Chen, 2024). These findings highlight the importance of supportive environments that balance competition and autonomy to sustain student motivation (Bandura, 1989).

#### 5.2.2. Effects of Competitive Environment

Building on our examination of the cognitive adaptation model, we now turn to environmental influences. The competitive environment significantly influenced competitive attitude at both levels (H_4w_: *β* = 0.206, *p* < 0.001; H_4b_: *β* = 0.708, *p* < 0.001), supporting H_4_. This aligns with research indicating that exposure to competitive environments fosters higher performance-driven attitudes (H. Wang et al., 2018; Mayor et al., 2020). However, its impact on task motivation varied significantly between the two levels (H_5w_: *β* = 0.050, *p* < 0.001; H_5b_: *β* = −0.573, *p* < 0.01), indicating that H_5_ is not fully consistent across levels. At the student level, competition enhanced task motivation, reinforcing self-determination theory (Lamb et al., 2019). However, at the school level, competition negatively influenced motivation, suggesting that excessive institutional pressure may hinder students’ willingness to engage in learning. This negative effect can be explained by the potential psychological strain that pervasive competition creates across the school environment, leading to diminished intrinsic interest and autonomous regulation (Ooi & Cortina, 2023; Tan et al., 2023). These findings align with social–ecological systems theory, which emphasizes that macro-level stressors within the school climate can undermine students’ intrinsic motivation. School policies should thus aim to moderate competition to prevent adverse psychological effects on students.

#### 5.2.3. Effects of Collaborative Environment

In contrast to competitive environments, the collaborative environment significantly influenced competitive attitude at the student level (H_6w_: *β* = 0.103, *p* < 0.001) but was not significant at the school level (H_6b_: *β* = 0.146, *p* = 0.092), indicating that H_6_ is partially supported. This supports the idea that collaborative environments shape students’ perceptions of competition at the student level but may not necessarily influence broader institutional attitudes (Ooi & Cortina, 2023). On the other hand, collaborative environments strongly enhanced task motivation at both levels (H_7w_: *β* = 0.180, *p* < 0.001; H_7b_: *β* = 0.623, *p* < 0.001), supporting H_7_. This finding aligns with goal orientation theory, which posits that collaborative learning fosters mastery goals and intrinsic motivation (Kaplan & Patrick, 2016; C. Yang et al., 2021; Stasielowicz, 2019). Prior research highlights the positive role of cooperative learning in increasing engagement and self-efficacy (Liu & Lipowski, 2021; Mendo-Lázaro et al., 2021). These results emphasize the need for educational institutions to foster collaboration as a means of improving motivation and adaptability.

### 5.3. Methodological Reflections

This study contributes methodologically by employing multilevel structural equation modeling (MSEM), which provides deeper insights into hierarchical effects often overlooked in traditional SEM. The findings reinforce Bronfenbrenner’s (1979) multilevel systems perspective, demonstrating that student-level and school-level interactions contribute to cognitive adaptability and motivation. The model exhibited strong overall fit indices, including CFI, TLI, RMSEA, and student-level (Within) SRMR. However, the school-level (Between) SRMR (0.159) exceeded conventional thresholds (typically < 0.08), suggesting potential misspecification at the school level. This limitation may stem from the complexity of modeling school-level constructs with limited clusters. Further refinements—such as enhancing between-level factor structures or increasing the number of clusters—could improve model precision (Muthén, 1994). Additionally, the elevated between-level SRMR may reflect genuine variability in how school environments influence student outcomes, indicating a need for more nuanced modeling approaches. Future research should explore alternative parameter constraints or school-specific moderating variables to optimize multilevel modeling in educational contexts.

### 5.4. Implications for Model Selection and Educational Practice

The findings highlight the significant limitations of single-level SEM models, which fail to capture cross-level variations in four critical ways. First, the effects of competitive attitude and task motivation on cognitive adaptability were significantly stronger at the school level, indicating that school-wide structures influence adaptive learning beyond individual experiences. Second, the positive relationship between competitive attitude and task motivation was no longer present at the school level, suggesting that school-wide competition does not necessarily translate into higher motivation. Third, the competitive environment had a negative influence on task motivation at the school level, confirming that excessive institutional competition may suppress student engagement. Finally, while the collaborative environment did not significantly affect competitive attitudes at the school level, its positive influence on task motivation remained strong, underscoring the role of school culture in fostering motivation (Turner et al., 2015; Moeyaert et al., 2014). These findings have practical implications for educational institutions. Schools should design environments that balance competition and collaboration, recognizing that while individual competition may motivate students, school-wide competitive climates might undermine collective motivation. Educational leaders should implement collaborative practices that enhance task motivation while carefully monitoring competitive elements to ensure they do not create undue pressure. By integrating multilevel SEM, this study provides a more precise and contextually relevant understanding of student motivation and adaptability, reinforcing the necessity of hierarchical analytical approaches in educational research and practice.

## 6. Conclusions

This study utilized multilevel structural equation modeling (MSEM) to analyze the mechanisms through which competitive and collaborative environments influence the cognitive adaptability of vocational students. The key findings are as follows.

### 6.1. Research Conclusions

First, from a theoretical perspective, this study successfully integrates social cognitive theory and social–ecological systems theory to construct a multilevel analytical framework. This framework systematically reveals the complex interrelationships between school environments, student cognitive factors, and adaptability. By capturing cross-level interactions, our analysis identifies significant differences that traditional single-level approaches cannot detect, offering a more comprehensive theoretical foundation for understanding cognitive adaptability development in vocational education.

Second, in addressing our first research question, we found that at the student level, both competitive attitude and task motivation significantly enhance cognitive adaptability. These relationships manifest within the overall model structure, with task motivation demonstrating a substantially stronger influence than competitive attitude. This suggests that fostering intrinsic motivation in vocational students is more critical for promoting cognitive adaptability than emphasizing competitiveness.

Third, our examination of the second research question revealed contrasting effects of competitive and collaborative environments at the school level. While student-level competition may benefit student motivation, a competitive school environment negatively impacts overall task motivation. In contrast, collaborative environments consistently exhibit positive effects on motivation. These findings highlight a critical educational implication: an overly competitive school atmosphere can harm collective motivation, whereas fostering a collaborative school culture more effectively enhances school-wide motivation and engagement.

Finally, our multilevel analysis results directly respond to the third research question by disentangling the differences between student- and school-level influences. At the school level, the effects of competitive attitude and task motivation on cognitive adaptability were considerably stronger than at the student level. This suggests that collective school-level influences can significantly amplify or suppress student-level effects. Traditional single-level analysis would fail to capture these nuanced cross-level relationships, demonstrating the necessity of multilevel modeling in educational research. These findings provide valuable insights for future policy development and instructional practices in vocational education, emphasizing the importance of cultivating positive school environments that balance individual competition with institutional collaboration.

### 6.2. Limitations

This study has several methodological limitations that should be acknowledged. First, the use of self-reported questionnaires may have introduced social desirability bias, potentially affecting the accuracy of the participants’ responses. Second, vocational education systems vary across countries, which poses challenges in cross-national comparisons. Therefore, caution is needed when interpreting the results. Third, according to the analysis results, the school-level (Between) SRMR (standardized root mean square residual) value exceeded the commonly accepted threshold. While this does not necessarily indicate an error in model specification (Rappaport et al., 2020), it suggests that improvements at the school level could enhance model fit. Additionally, among the key variables in this study, cognitive adaptability was measured using a five-point scale, whereas all other variables used a four-point Likert scale. To mitigate the impact of differences in the measurement scales, standardized coefficients were applied, improving comparability across different instruments (Schweizer, 2011; Wen et al., 2010; C. H. Li, 2021). Furthermore, when the sample size is sufficiently large, the MLR (maximum likelihood robust) estimation method can improve estimation accuracy (Rhemtulla et al., 2012). Despite these efforts to minimize potential biases, this remains a limitation of the study.

### 6.3. Future Research Directions

For future research, studies employing multilevel structural equation modeling (MSEM) should directly conduct multilevel confirmatory factor analysis (MCFA) and multilevel structural equation modeling (MSEM) as the primary analytical steps. Researchers should only revert to the five-step diagnostic procedure if the model fails to converge. This approach streamlines the analysis process and enhances efficiency while ensuring robust model estimation. Given the complexity of multilevel models and the limitations of the MLR method, future studies may consider Bayesian estimation to improve model convergence and stability. Furthermore, even in studies that initially seem appropriate for single-level analysis, researchers should prioritize multilevel modeling whenever possible. This allows better control of between-level influences, yielding more precise parameter estimates than traditional single-level models. It also prevents the omission of hierarchical effects, thereby enhancing the accuracy and interpretability of the findings. By implementing these methodological refinements, future research can achieve a more comprehensive understanding of the interactions between individuals, groups, and systems in education and social sciences. These advancements will contribute to both theoretical development and practical applications, providing a stronger empirical foundation for future studies in these fields.

## Figures and Tables

**Table 1:** Research hypotheses and their theoretical support.

Hyp.	Path	Supporting Literature
Social Cognitive Theory		(Bandura, 1986, 1989; Schunk & DiBenedetto, 2020)
H_1_	Attitude → Adapt	(Ding et al., 2023; H. Wang et al., 2018)
H_2_	Attitude → Motivate	(Ma & Chen, 2024)
H_3_	Motivate → Adapt	(Schunk & DiBenedetto, 2020; Abdelrahman, 2020; Naamati-Schneider & Alt, 2023; Chuang et al., 2022)
Social–Ecological Systems Theory		(Bronfenbrenner, 1977, 1979)
H_4_	CompEnv → Attitude	(Ma & Chen, 2024; H. Wang et al., 2018; Mayor et al., 2020)
H_5_	CompEnv → Motivate	(Ma & Chen, 2024; Ooi & Cortina, 2023; Lamb et al., 2019; Tan et al., 2023)
H_6_	CollEnv → Attitude	(Ooi & Cortina, 2023)
H_7_	CollEnv → Motivate	(Kaplan & Patrick, 2016; C. Yang et al., 2021; Liu & Lipowski, 2021; Mendo-Lázaro et al., 2021; Naamati-Schneider & Alt, 2023)

Note: Hyp. = hypothesis; Adapt = cognitive adaptability; Attitude = competitive attitude; Motivate = task motivation; CompEnv = competitive environment; CollEnv = collaborative environment.

**Table 2:** Construct reliability and validity analysis.

	Conventional CFA				Multilevel CFA					
Factor	Single Level			ICC	Within Level (Student Level)			Between Level (School Level)		
Item	Estimate	CR	AVE		Estimate	CR	AVE	Estimate	CR	AVE
1. Adapt: Cognitive adaptability										
Adapt1	0.687	0.866	0.520	0.062	0.687	0.863	0.514	0.808	0.909	0.632
Adapt2	0.695			0.025	0.688			0.610		
Adapt3	0.716			0.026	0.756			0.788		
Adapt4	0.708			0.024	0.680			0.566		
Adapt5	0.764			0.040	0.761			0.939		
Adapt6	0.753			0.046	0.724			0.972		
2. Attitude: Competitive Attitude										
Attitude1	0.762	0.832	0.623	0.056	0.711	0.785	0.550	0.581	0.821	0.614
Attitude2	0.781			0.075	0.739			0.946		
Attitude3	0.824			0.042	0.773			0.781		
3. Motivate: Task Motivation										
Motivate1	0.746	0.808	0.518	0.087	0.719	0.807	0.513	0.734	0.853	0.598
Motivate2	0.757			0.024	0.735			0.678		
Motivate3	0.811			0.065	0.776			0.971		
Motivate4	0.534			0.058	0.626			0.671		
4. CompEnv: Competitive Environment										
CompEnv1	0.810	0.894	0.680	0.055	0.741	0.867	0.622	0.938	0.940	0.799
CompEnv2	0.900			0.046	0.861			0.928		
CompEnv3	0.861			0.049	0.850			0.975		
CompEnv4	0.717			0.030	0.690			0.711		
5. CollEnv: Collaborative Environment										
CollEnv1	0.836	0.933	0.777	0.056	0.820	0.923	0.750	0.973	0.993	0.972
CollEnv2	0.902			0.062	0.892			0.998		
CollEnv3	0.920			0.054	0.907			0.996		
CollEnv4	0.865			0.046	0.842			0.977		

Note: CR: composite reliability; AVE: average variance extracted; ICC: intraclass correlation coefficient. All estimates represent standardized coefficients.

**Table 3:** Construct discriminant validity analysis.

**1. Conventional CFA (Single Level) Discriminant Validity Analysis**							
	Factor	AVE	1. Adapt	2. Attitude	3. Motivate	4. CompEnv	5. CollEnv
	1. Adapt	0.520	0.721				
	2. Attitude	0.623	0.201	0.789			
	3. Motivate	0.518	0.243	0.550	0.720		
	4. CompEnv	0.680	0.161	0.237	0.205	0.825	
	5. CollEnv	0.777	0.195	0.161	0.256	0.281	0.881
**2. Multilevel CFA Discriminant Validity Analysis**							
**2-1**	**Within level (Student Level)**						
	Factor	AVE	1. _w_Adapt	2. _w_Attitude	3. _w_Motivate	4. _w_CompEnv	5. _w_CollEnv
	1. _w_Adapt	0.514	0.717				
	2. _w_Attitude	0.550	0.167	0.742			
	3. _w_Motivate	0.513	0.251	0.494	0.716		
	4. _w_CompEnv	0.622	0.126	0.230	0.194	0.789	
	5. _w_CollEnv	0.750	0.172	0.150	0.256	0.233	0.866
**2-2**	**Between level (School Level)**						
	Factor	AVE	1. _b_Adapt	2. _b_Attitude	3. _b_Motivate	4. _b_CompEnv	5. _b_CollEnv
	1. _b_Adapt	0.632	0.795				
	2. _b_Attitude	0.614	0.126	0.784			
	3. _b_Motivate	0.598	0.347	0.050	0.773		
	4. _b_CompEnv	0.799	0.326	0.683	−0.114	0.894	
	5. _b_CollEnv	0.972	0.335	0.444	0.406	0.535	0.986

Note: (1) Adapt: cognitive adaptability; Attitude: competitive attitude; Motivate: task motivation; CompEnv: competitive environment; CollEnv: collaborative environment. (2) For readability, “w” represents within level (student level); “b” represents between level (school level). (3) The bold diagonal values represent the square roots of average variance extracted (AVE). (4) The lower triangular matrix contains the Pearson correlation coefficients.

**Table 4:** Overall structural model parameter estimation table.

Conventional SEM Path Analysis (Single Level)						
Hypotheses	Path	Standardized Est.	Unstandardized Est.	S.E.	Est./S.E.	*p*-Value
H_1_	Attitude → Adapt	0.095	0.091	0.021	4.389	***
H_2_	Attitude → Motivate	0.514	0.443	0.019	23.306	***
H_3_	Motivate → Adapt	0.198	0.219	0.028	7.941	***
H_4_	CompEnv → Attitude	0.210	0.203	0.018	11.226	***
H_5_	CompEnv → Motivate	0.039	0.033	0.014	2.348	0.019
H_6_	CollEnv → Attitude	0.104	0.098	0.018	5.337	***
H_7_	CollEnv → Motivate	0.166	0.134	0.014	9.794	***
**Multilevel SEM Path Analysis**						
**Within level** (Student Level)						
H_1w_	_w_Attitude → _w_Adapt	0.053	0.057	0.013	4.449	***
H_2w_	_w_Attitude → _w_Motivate	0.455	0.409	0.012	34.682	***
H_3w_	_w_Motivate → _w_Adapt	0.231	0.277	0.015	18.098	***
H_4w_	_w_CompEnv → _w_Attitude	0.206	0.205	0.010	20.478	***
H_5w_	_w_CompEnv → _w_Motivate	0.050	0.045	0.009	5.143	***
H_6w_	_w_CollEnv → _w_Attitude	0.103	0.093	0.009	10.070	***
H_7w_	_w_CollEnv → _w_Motivate	0.180	0.147	0.008	19.009	***
**Between level** (School Level)						
H_1b_	_b_Attitude → _b_Adapt	0.340	0.465	0.158	2.943	0.003
H_2b_	_b_Attitude → _b_Motivate	0.177	0.214	0.247	0.866	0.386
H_3b_	_b_Motivate → _b_Adapt	0.333	0.376	0.075	5.037	***
H_4b_	_b_CompEnv → _b_Attitude	0.708	0.539	0.091	5.899	***
H_5b_	_b_CompEnv → _b_Motivate	−0.573	−0.528	0.163	−3.241	0.001
H_6b_	_b_CollEnv → _b_Attitude	0.146	0.107	0.064	1.683	0.092
H_7b_	_b_CollEnv → _b_Motivate	0.623	0.554	0.068	8.105	***

Note: (1) Adapt: cognitive adaptability; Attitude: competitive attitude; Motivate: task motivation; CompEnv: competitive environment; CollEnv: collaborative environment. (2) For readability “w” denotes within level (student level), and “b” denotes between level (school level). (3) S.E. = standard error; Est./S.E. = estimate divided by standard error. (4) *** *p* < 0.001.

## Data Availability

This study analyzes existing public data from the PISA 2018 (Programme for International Student Assessment) dataset. The data is freely available through the OECD website (https://www.oecd.org/en/data/datasets/pisa-2018-database.html, accessed on 7 February 2023). The dataset includes student assessment results, questionnaire data, and technical documentation. No new data were created in this study.

## Appendix A

PISA 2018 student questionnaire items and corresponding item numbers.

Item	Question Content
1. CA: Cognitive adaptability (Code in dataset: ST216) How well does each of the following statements below describe you?	
CA1	I can deal with unusual situations.
CA2	I can change my behavior to meet the needs of new situations.
CA3	I can adapt to different situations even when under stress or pressure.
CA4	I can adapt easily to a new culture.
CA5	When encountering difficult situations with other people, I can think of a way to resolve the situation.
CA6	How well does the following describe you: I am capable of overcoming my difficulties in interacting with people from other cultures.
2. AT: Competitive Attitude (Code in dataset: ST181) How much do you agree with the following statements about yourself?	
AT1	I enjoy working in situations involving competition with others.
AT2	It is important for me to perform better than other people on a task.
AT3	I try harder when I’m in competition with other people.
3. MT: Task Motivations (Code in dataset: ST182) How much do you agree with the following statements about yourself?	
MT1	I find satisfaction in working as hard as I can.
MT2	Once I start a task, I persist until it is finished.
MT3	Part of the enjoyment I get from doing things is when I improve on my past performance.
MT4	If I am not good at something, I would rather keep struggling to master it than move on to something I may be good at.
4. CM: Competitive Environment (Code in dataset: ST205) Think about your school: how true are the following statements?	
CM1	Students seem to value competition.
CM2	It seems that students are competing with each other.
CM3	Students seem to share the feeling that competing with each other is important.
CM4	Students feel that they are being compared with others.
5. CO: Collaborative Environment (Code in dataset: ST206) Think about your school: how true are the following statements?	
CO1	Students seem to value cooperation.
CO2	It seems that students are cooperating with each other.
CO3	Students seem to share the feeling that cooperating with each other is important.
CO4	Students feel that they are encouraged to cooperate with others.

## Appendix B

*Descriptive Analysis* (**Vocational Schools** *N* = 814; **Vocational Students** *N* = 20,978).

**1. Vocational Schools**			**2. Vocational Students**		
**Country**	**n**	**%**	**Country**	**n**	**%**
(1) Belarus	13	1.6	(1) Belarus	342	1.6
(2) Chile	27	3.3	(2) Chile	569	2.7
(3) Chinese Taipei	41	5.0	(3) Chinese Taipei	1409	6.7
(4) Costa Rica	45	5.5	(4) Costa Rica	1362	6.5
(5) Croatia	104	12.8	(5) Croatia	2557	12.2
(6) France	35	4.3	(6) France	486	2.3
(7) Hungary	87	10.7	(7) Hungary	1894	9.0
(8) Ireland	45	5.5	(8) Ireland	1080	5.1
(9) Korea	26	3.2	(9) Korea	867	4.1
(10) Macao	5	0.6	(10) Macao	114	0.5
(11) Malaysia	8	1.0	(11) Malaysia	242	1.2
(12) Montenegro	26	3.2	(12) Montenegro	2414	11.5
(13) Poland	2	0.2	(13) Poland	6	0.0
(14) Serbia	32	3.9	(14) Serbia	611	2.9
(15) Slovenia	177	21.7	(15) Slovenia	2815	13.4
(16) Turkey	56	6.9	(16) Turkey	1815	8.7
(17) North Macedonia	51	6.3	(17) North Macedonia	2073	9.9
(18) Uruguay	34	4.2	(18) Uruguay	322	1.5
**Total**	814	100.0	**Total**	20,978	100.0
**3. Gender Distribution**			**4. Grade Distribution**		
**Gender**	**n**	**%**	**Grade**	**n**	**%**
(1) Female	9500	45.3	(1) Grade 7	73	0.3
(2) Male	11,478	54.7	(2) Grade 8	238	1.1
**Total**	20,978	100.0	(3) Grade 9	7887	37.6
			(4) Grade 10	12,459	59.4
			(5) Grade 11	320	1.5
			(6) Grade 12	1	0.0
			**Total**	20,978	100.0

## Appendix C. Overall Structural Model Parameter Estimation Table

**Table:**

Model	χ^2^	*df*	*p*	CFI	TLI	RMSEA	SRMR
1. Conventional CFA	1260.681	179	***	0.972	0.967	0.017	0.021
2. Multilevel CFA	5938.011	358	***	0.968	0.963	0.027	Within 0.022 Between 0.168
3. Conventional SEM	1339.685	181	***	0.970	0.965	0.017	0.037
4. Multilevel SEM	6216.432	362	***	0.967	0.962	0.028	Within 0.033 Between 0.159

Note: *** *p* < 0.001.

## Appendix D. Mplus Syntax for Multilevel SEM (PISA 2018)

This appendix presents the Mplus 8.10 syntax for multilevel structural equation modeling with PISA 2018 data. The syntax accounts for complex sampling (weights, clustering, stratification) to enhance statistical accuracy. These examples support replication and future research.

TITLE:
Multilevel SEM with Complex Sampling

DATA:
FILE = WPISA_VOC.csv; ! Input data file

VARIABLE:
NAMES = SCID STRATUM W_FSTUWT AT1-AT3 MT1-MT4 CA1-CA6 CM1-CM4 CO1-CO4;
USEVARIABLES = AT1-AT3 MT1-MT4 CA1-CA6 CM1-CM4 CO1-CO4;

! Variable Definitions:
! AT: Competitive Attitude
! MT: Task Motivation
! CA: Cognitive Adaptability
! CM: Competitive Environment
! CO: Collaborative Environment

WEIGHT = W_FSTUWT; ! Student-level sampling weight
CLUSTER = SCID; ! School-level clustering variable
STRATIFICATION = STRATUM; ! School stratification variable

ANALYSIS:
ESTIMATOR = MLR; ! Maximum Likelihood Estimation with Robust Standard Errors
TYPE = TWOLEVEL COMPLEX; ! Multilevel SEM with complex sampling design
H1ITERATIONS = 100000; ! Maximum iterations for H1 model convergence
PROCESS = 4; ! Use 4 CPU cores for faster computation

MODEL:

! -------------------------
! Default: Multilevel Confirmatory Factor Analysis (MCFA)
! -------------------------

%WITHIN% ! Student-Level
WAT by AT1-AT3;
WMT by MT1-MT4;
WCA by CA1-CA6;
WCM by CM1-CM4;
WCO by CO1-CO4;

%BETWEEN% ! School-Level
BAT by AT1-AT3;
BMT by MT1-MT4;
BCA by CA1-CA6;
BCM by CM1-CM4;
BCO by CO1-CO4;

! -------------------------
! Optional: Multilevel Structural Model Testing (MSEM)
! To enable MSEM, uncomment the following lines
! -------------------------

!%WITHIN%
!WCA on WAT WMT; ! H1w H3w
!WAT on WCM WCO; ! H4w H6w
!WMT on WAT WCM WCO; ! H2w H5w H7w

!%BETWEEN%
!BCA on BAT BMT; ! H1b H3b
!BAT on BCM BCO; ! H4b H6b
!BMT on BAT BCM BCO; ! H2b H5b H7b

OUTPUT:
SAMPSTAT STDYX;
